# Diagnostic and prognostic utility of cardiovascular magnetic resonance imaging in heart failure with preserved ejection fraction – implications for clinical trials

**DOI:** 10.1186/s12968-017-0424-9

**Published:** 2018-01-11

**Authors:** Prathap Kanagala, Adrian S. H. Cheng, Anvesha Singh, John McAdam, Anna-Marie Marsh, Jayanth R. Arnold, Iain B. Squire, Leong L. Ng, Gerry P. McCann

**Affiliations:** 1 Department of Cardiovascular Sciences and National Institute for Health Research (NIHR), University of Leicester, Leicester Cardiovascular Biomedical Research Unit, Glenfield Hospital, Groby Road, Leicester, LE3 9QP UK; 2 Kettering General Hospital, Kettering, UK; 3 National Institute for Health Research (NIHR) Leicester Cardiovascular Biomedical Research Unit, Glenfield Hospital, Leicester, UK; 4 Department of Cardiovascular Sciences and National Institute for Health Research (NIHR), University of Leicester, Leicester Cardiovascular Biomedical Research Unit, Cardiovascular Medicine, Glenfield Hospital, Leicester, UK; 5 Department of Cardiovascular Sciences and National Institute for Health Research (NIHR), Medicine and Therapeutics, University of Leicester, Leicester Cardiovascular Biomedical Research Unit, Glenfield Hospital, Leicester, UK; 6 Department of Cardiovascular Sciences and National Institute for Health Research (NIHR), Cardiac Imaging, University of Leicester, Leicester Cardiovascular Biomedical Research Unit, Glenfield Hospital, Leicester, UK

**Keywords:** Cardiovascular magnetic resonance imaging, Heart failure, Heart failure with preserved ejection fraction, Diagnostic, Prognostic, Transthoracic echocardiography

## Abstract

**Background:**

Heart failure with preserved ejection fraction (HFpEF) is a poorly characterized condition. We aimed to phenotype patients with HFpEF using multiparametric stress cardiovascular magnetic resonance imaging (CMR) and to assess the relationship to clinical outcomes.

**Methods:**

One hundred and fifty four patients (51% male, mean age 72 ± 10 years) with a diagnosis of HFpEF underwent transthoracic echocardiography and CMR during a single study visit. The CMR protocol comprised cine, stress/rest perfusion and late gadolinium enhancement imaging on a 3T scanner. Follow-up outcome data (death and heart failure hospitalization) were captured after a minimum of 6 months.

**Results:**

CMR detected previously undiagnosed pathology in 42 patients (27%), who had similar baseline characteristics to those without a new diagnosis. These diagnoses consisted of: coronary artery disease (*n* = 20, including 14 with ‘silent’ infarction), microvascular dysfunction (*n* = 11), probable or definite hypertrophic cardiomyopathy (*n* = 10) and constrictive pericarditis (*n* = 5). Four patients had dual pathology. During follow-up (median 623 days), patients with a new CMR diagnosis were at higher risk of adverse outcome for the composite endpoint (log rank test: *p* = 0.047). In multivariate Cox proportional hazards analysis, a new CMR diagnosis was the strongest independent predictor of adverse outcome (hazard ratio: 1.92; 95% CI: 1.07 to 3.45; *p* = 0.03).

**Conclusions:**

CMR diagnosed new significant pathology in 27% of patients with HFpEF. These patients were at increased risk of death and heart failure hospitalization.

**Trial registration:**

ClinicalTrials.gov Identifier: NCT03050593. Retrospectively registered; Date of registration: February 06, 2017.

## Background

Heart failure with preserved ejection fraction (HFpEF) presents with marked clinical heterogeneity and accounts for approximately half of all heart failure (HF) cases. It is projected to be the predominant phenotype in the near future [1, 2]. While treatments have improved outcomes in heart failure with reduced ejection fraction (HFrEF), similar therapies have not been shown to improve outcome in HFpEF and there remain no specific, evidence-based treatments [3]. Furthermore, a wide range of pathologies such as silent myocardial infarction (MI) and ischaemia due to coronary artery disease (CAD), hypertrophic cardiomyopathy (HCM) and constrictive pericarditis may masquerade as HFpEF [4–6]. These ‘phenocopies’ may share many features of HFpEF such as preserved ejection fraction (EF), left ventricular hypertrophy (LVH), diastolic dysfunction, atrial dilatation and elevated natriuretic peptides. Hence, focus has shifted to studying ‘purer’ forms of HFpEF by excluding such conditions from contemporary clinical trials [7].

Transthoracic echocardiography (TTE) remains the primary diagnostic tool for HFpEF [8] and the gatekeeper for entry into clinical trials of this entity [3, 7]. However, cardiovascular magnetic resonance imaging (CMR) is the recognized gold standard for assessment of the majority of parameters that make up the latest HFpEF guidelines [8–11]. The superior diagnostic capabilities of CMR across the spectrum of aforementioned ‘phenocopies’ is also well established [5, 12–14]. However, no reports in the literature detail the systematic use of CMR in patients with suspected HFpEF. We aimed to establish the proportion of new clinical diagnoses in HFpEF patients identified with CMR, and to assess their impact upon clinical outcome.

## Methods

### Study population

Patients were recruited as part of an observational cohort study conducted at a single tertiary cardiac centre. The inclusion criteria were: clinical or radiographic evidence of HF, EF > 50% on transthoracic echocardiography (TTE) and age ≥ 18 years. The exclusion criteria were: MI in the preceding 6 months, suspected or confirmed cardiomyopathy or constrictive pericarditis, non-cardiovascular life expectancy <6 months, severe native valve disease, severe chronic obstructive pulmonary disease (or forced expiratory volume [FEV_1_] < 30% predicted or forced vital capacity [FVC] <50% predicted) and estimated glomerular filtration rate (eGFR) < 30 ml/min per 1.73m^2^. The study was approved by the National Research Ethics Service. All subjects provided written informed consent prior to participation.

Potentially eligible patients were invited to participate following screening of the hospital database, outpatient clinics and wards. All enrolled patients underwent comprehensive clinical assessment (including patient reporting of angina symptoms and previous MI or revascularization), venepuncture, 12-lead electrocardiography (ECG) and TTE followed by CMR (provided no contraindications) during the same visit. The clinical reports of all scans were disseminated to the responsible physician(s) to inform patient management.

### Blood samples

Blood was sampled for B-type natriuretic peptide (BNP) immunoassay (Siemens, Erlangen, Germany) and other biochemical markers (sodium, urea and creatinine). Estimated GFR was calculated from the Modification of Diet in Renal Disease formula.

### ECG

The 12-lead ECGs performed were assessed (by PK and AMM) for the presence of pathological Q waves as surrogates of transmural MI [15].

### Imaging

Clinical reports were generated for TTE and CMR scans with knowledge of patient demographics and past medical history (e.g. history of hypertension). All subsequent quantitative and qualitative analyses used to generate the reports were performed independently with readers blinded to data from the other scan. Image quality was graded as: 0 = non-interpretable; 1 = poor; 2 = fair; 3 = good.

### TTE

TTE studies were performed and reported by two accredited sonographers (AMM, JM). Images were acquired and reported as per American Society of Echocardiography guidelines using an iE 33 system with S5–1 transducer (Philips Medical Systems, Best, The Netherlands) [16]. Left ventricular (LV) EF for study inclusion was calculated using the biplane method or estimated visually in cases of poor endocardial border definition. For borderline cases, final consensus required review by a third observer (PK). Any regional wall motion abnormalities (RWMA) were reported.

### CMR

CMR scans were performed on a 3T scanner (Siemens Skyra Erlangen, Germany) with an 18-channel cardiac coil. The protocol was previously reported by our group [17]. Cine imaging was performed in three long axes and a short axis cine stack was performed in the interval between stress and rest perfusion acquisitions. For pharmacological stress, 140–210 mcg/kg/min adenosine (depending on haemodynamic and symptomatic response) was infused for at least 3 min. Stress and rest perfusion images at the basal, mid-ventricular and apical levels were acquired after injection of 0.04 mmol/kg of contrast (Gadovist, Bayer Healthcare, Berlin, Germany). Following rest perfusion, a ‘top-up’ bolus of 0.07 mmol/kg was given to make a total contrast dose of 0.15 mmol/kg. Late gadolinium enhancement imaging (LGE) was performed 10–15 min after the final injection of contrast.

CMR analyses and reporting were undertaken with cases randomly split between two experienced imaging cardiologists (GPM, ASHC). LV EF and volumes, wall thickness and perfusion were assessed using commercially available software (Argus, Siemens Healthineers, Erlangen, Germany). LV contours were drawn manually (excluding papillary muscles) to derive end-diastolic and end-systolic volumes and LVEF from the short-axis cine stack as reported by our group previously with excellent intra-observer and inter-observer variability [18]. Volumetric data were indexed to body surface area.

### Definitions of ‘new diagnoses’ from CMR

MI was defined as high signal intensity area(s) on LGE involving at least the sub-endocardium in a coronary artery distribution and the segmental extent and transmurality were described. For ischaemia evaluation, in conjunction with LGE images, stress and rest perfusion images were semi-quantitatively assessed for reversible perfusion defects. The defects were categorized into ischaemia likely to be due to epicardial CAD or microvascular dysfunction [19]. Ischaemia was defined by the identification of inducible perfusion defects as per published Society for Cardiovascular Magnetic Resonance guidance [19]. Circumferential, sub-endocardial perfusion defects seen at least on one ventricular level or crossing coronary territories were reported as suggestive of microvascular dysfunction, albeit with the caveat that significant CAD could not be reliably excluded.

Constrictive pericarditis (e.g. diastolic septal bounce, pericardial effusion, thickening and hyperenhancement on LGE) and HCM were diagnosed based on established CMR parameters [5, 12, 14, 20]. A diagnosis of HCM was considered in all patients with LV wall thickness of ≥15 mm [12]. In such cases, the degree and pattern of LVH and medical history (including hypertension, blood pressure control, anti-hypertensive medications) were considered to gauge whether wall thickness was proportionate or disproportionate. A characteristic spade-like configuration of the LV cavity and apical:basal wall thickness ratio ≥ 1.3 was used to diagnose apical HCM [14]. The overall likelihood of HCM was categorized as definite or probable.

### Follow-up and endpoints

Patients were followed up for a minimum of 6 months post-study entry. The primary endpoint was the combination of hospitalization for HF (defined as a hospital admission for which HF was the primary reason and which required diuretic, inotropic or intravenous nitrate therapy) or all-cause mortality. Hospital databases and patient records were sourced to obtain outcome data.

### Statistical analysis

Statistical analyses were performed using SPSS (version 22, International Business Machines, Inc., Armonk, New York, USA). Probability (p) values <0.05 were considered statistically significant. Normality was assessed using the Shapiro-Wilk test, histograms and Q-Q plots. Normally distributed data are expressed as mean ± SD. Non-parametric data are expressed as median (25–75% interquartile range [IQR]). Categorical data are expressed as absolute numbers or percentages. Comparisons of means of 2 groups were performed using the independent samples *t* test. The chi-square test was used to compare categorical data. Cohen’s Kappa (Κ) was used to test for agreements of similarities in image grading between CMR and TTE (*p* > 0.05 was considered significant). Cox proportional hazard and multiple regression analyses were performed to determine which variables were related significantly to the composite endpoint of death and/or hospitalization with HF. BNP levels were log_10_ transformed and hazard ratios for subsequent analysis refer to 1 standard deviation (SD) increment of the transformed BNP. Only variables with a univariate *p* value <0.10 were entered into subsequent multivariate analysis. Kaplan-Meier survival curves were used to demonstrate cumulative event-free rates in patients stratified into 2 CMR groups (‘no new diagnoses’ versus ‘new diagnoses’) and a log rank test was used to test for statistical significance.

## Results

**Fig. 1:**
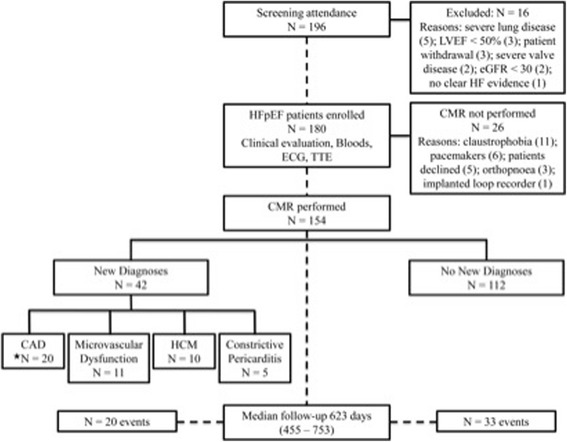
Study overview. *Of the 20 patients with newly diagnosed coronary artery disease (CAD), 4 patients had concomitant hypertrophic cardiomyopathy (HCM)

A summary of the study overview, patients excluded and results are presented in Fig. 1. One hundred and ninety six patients attended for screening. Severe lung disease was the most common reason for exclusion. One hundred and eighty patients met the initial study inclusion criteria. The majority of patients who did not undergo subsequent CMR evaluation were either claustrophobic or had pacemakers.

A total of 154 patients underwent CMR, of whom 5 did not undergo stress perfusion imaging. Baseline characteristics of the CMR population stratified by the presence or absence of new CMR diagnoses, are summarized in Table 1. Patients with and without new diagnoses on CMR had similar baseline characteristics, including LV volumes and LVEF. The cohort had a wide age range (37–97 years) with the majority of patients over 65 years. Nearly one-third were in NYHA class III or IV. There was a high prevalence of obesity and hypertension and nearly half the patients had a history of atrial fibrillation and a similar proportion of diabetes. Approximately a fifth had chronic lung disease. The proportion of patients with CAD at baseline was 21%, including 15 patients with known MI.

**Table 1:** Baseline characteristics of the patients who underwent CMR

	All	No new diagnoses group (*n* = 112)	New diagnoses group (*n* = 42)	*p* value
*Demographics*				
Age, years	72 ± 10	73 ± 9	72 ± 12	0.61
Male	78 (50.6)	54 (48.2)	24 (57.1)	0.32
*Clinical findings*				
Atrial fibrillation	72 (46.8)	50 (44.6)	24 (52.4)	0.42
Heart rate (bpm)	70 ± 14	70 ± 14	72 ± 16	0.57
Systolic blood pressure (mmHg)	143 ± 25	144 ± 25	146 ± 26	0.61
Diastolic blood pressure (mmHg)	74 ± 12	74 ± 12	74 ± 13	0.99
Body mass index (kg/m^2^)	34 ± 7	34 ± 7	33 ± 9	0.66
NYHA				
I/II	106 (68.8)	77 (68.8)	29 (69.0)	0.97
III/IV	48 (31.2)	35 (31.3)	13 (31.0)	
*Medical History*				
Known coronary artery disease	32 (20.8)	–	–	–
Hypertension	139 (90.3)	111 (89.3)	39 (92.9)	0.60
Diabetes	75 (48.7)	54 (48.2)	21 (50.0)	0.88
Chronic obstructive pulmonary disease or asthma	27 (17.5)	17 (15.2)	10 (23.8)	0.21
*Chest radiography*				
Pulmonary edema	110 (71.4)	79 (70.5)	31 (73.8)	0.69
*Medication*				
Aspirin	54 (35.1)	42 (37.5)	12 (28.6)	0.30
Beta-blocker	99 (64.3)	74 (66.1)	25 (59.5)	0.45
ACEi or ARB	130 (84.4)	97 (86.6)	33 (78.6)	0.22
Statin	97 (63.0)	70 (62.5)	27 (64.3)	0.84
Loop diuretic	125 (81.2)	91 (81.3)	34 (81.0)	0.97
*Biochemistry*				
Sodium (mmol/L)	139 ± 3.4	139 ± 3.6	140 ± 2.6	0.39
Urea (mmol/L)	8.7 ± 3.8	8.8 ± 4.0	8.3 ± 3.5	0.46
eGFR (ml/min per 1.73m^2^)	66 ± 19	66 ± 19	64 ± 19	0.46
BNP (ng/L, median, IQR)	145 (66–259)	134 ± (57.5–251)	175 ± (111–263)	*0.12
*CMR*				
LVEF (%)	57 ± 6	57 ± 6	57 ± 7	0.98
LVEDVI (ml/m^2^)	74 ± 18	73 ± 17	77 ± 21	0.26
LVESVI (ml/m^2^)	33 ± 11	32 ± 10	34 ± 13	0.30

Values are mean ± SD or n (%). The *p* values are for the t-test or chi-square test. *ACEi* angiotensin converting enzyme inhibitor; ARB = angiotensin II receptor blocker, *BNP* B-type natriuretic peptide, *CMR* cardiovascular magnetic resonance imaging, *eGFR* estimated glomerular filtration rate, *LVEF* left ventricular ejection fraction, *LVEDVI* left ventricular end-diastolic volume indexed to body surface area, *LVESVI* left ventricular end-systolic volume indexed to body surface area; * *p* value refers to zlog_10_ transformed BNP

### Imaging

Overall, image quality was better for CMR compared to TTE (median grade: 3 vs 2 respectively). In those with a new diagnosis on CMR, this difference was also maintained and statistically significant (kappa statistic [−0.021], *p* = 0.72).

### ‘New diagnoses’ from CMR

**Fig. 2:**
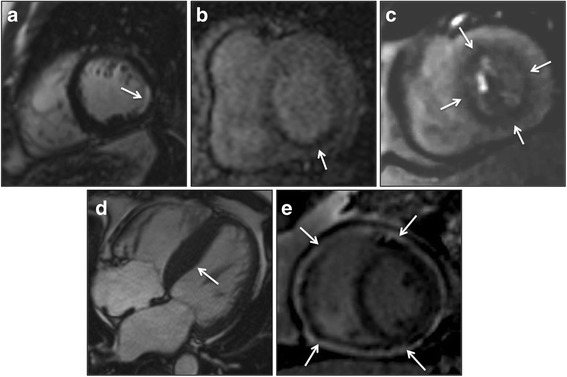
Examples of typical findings in the ‘new diagnoses’ group. CMR images of: **a** sub-endocardial, inferolateral myocardial infarction of 25–50% transmurality on LGE; **b** inferoseptal and inferior perfusion defect consistent with right coronary artery territory ischaemia; **c** global, concentric perfusion defect consistent with microvascular dysfunction; **d** horizontal long axis cine demonstrating asymmetrical septal hypertrophy in HCM; E) constrictive pericarditis with circumferential pericardial hyperenhancement on LGE; white arrows point towards pathology; LGE = late gadolinium enhancement imaging

CMR identified previously unknown diagnoses in 42 patients (27%). The following new pathologies (see Fig. 1) were noted: epicardial CAD based on MI or ischaemia (*n* = 20), microvascular dysfunction (*n* = 11), HCM (*n* = 10) and constrictive pericarditis (*n* = 5). Three patients with HCM had co-existent CAD (2 with new MI and 1 with ischaemia). One patient with constrictive pericarditis also had concurrent MI. Examples are shown in Fig. 2.

### CAD

**Fig. 3:**
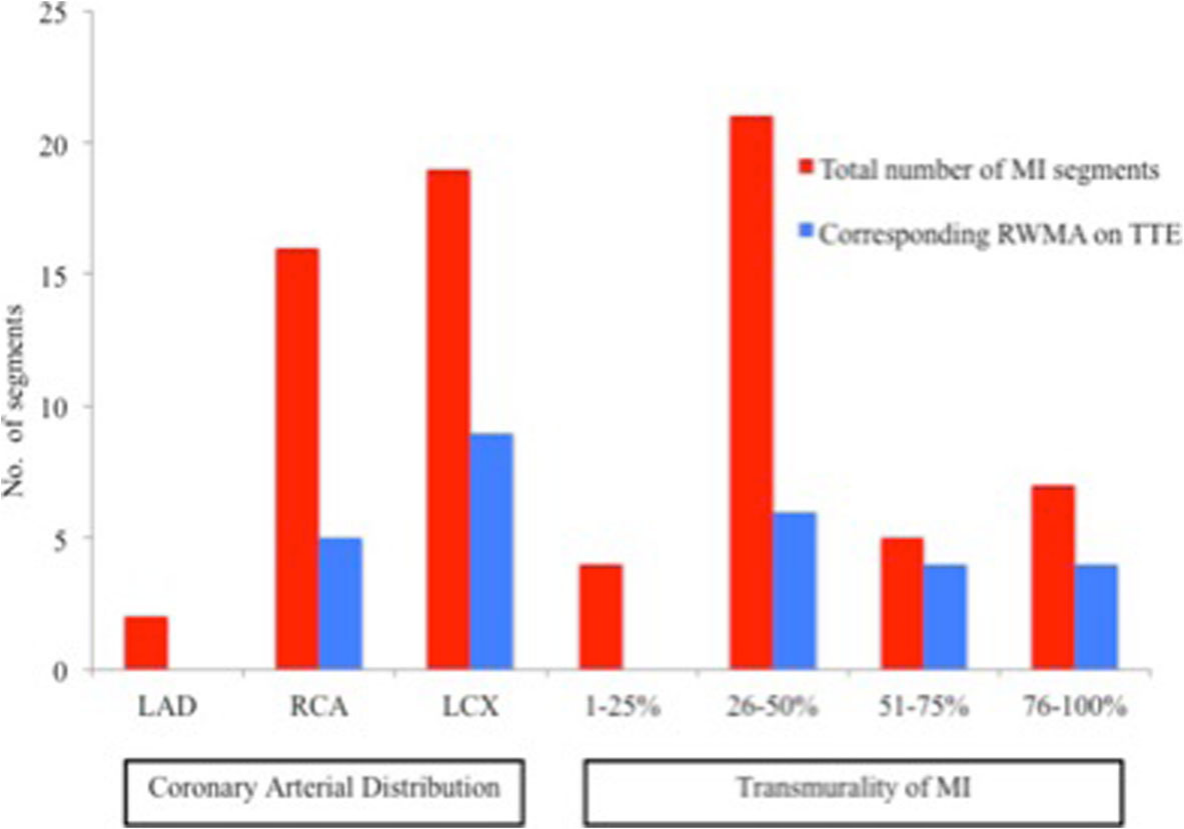
Characteristics of newly diagnosed myocardial infarction according to coronary arterial distribution and transmurality. LAD = left anterior descending artery; RCA = right coronary artery; LCX = left circumflex artery; % transmurality of MI is illustrated as 1–25, 26–50, 51–75, 76–100; RWMA = regional wall motion abnormality

Fourteen patients had LGE indicating ‘silent’ MI (affecting 37 segments). Of these, 3 patients had known CAD at baseline but no prior known MI or pathological Q waves on ECG. On segmental analysis (see Fig. 3), infarcts were typically small, in a territory not subtended by the left anterior descending coronary artery (95%) and of <50% transmurality (68%). Corresponding RWMAs on TTE were only reported in 38%. As expected, the ability to diagnose MI by regional wall motion abnormality (RWMA) detectable by TTE worsened with diminishing transmurality of MI (0–50% [24%] versus 51–100% [67%]). On review of the corresponding ECGs, only one case fulfilled the Q wave criterion for MI [15].

There were 31 patients with reversible perfusion defects and in 11 of these, the pattern suggested microvascular dysfunction. In the remaining 20 patients with ischaemia in an epicardial CAD pattern, 13 had no prior known CAD. Of these 13 patients, CMR detected a new diagnosis of MI in 4 patients. Accounting for these, CAD was newly diagnosed in 20 patients (new MI in 11 and new ischaemia in 9).

### HCM

Findings consistent with HCM (definite: *n* = 4; probable: *n* = 6) were reported in CMR studies of 10 patients. Individual patient characteristics are shown in Table 2. The main CMR phenotypic patterns of HCM were: asymmetrical septal hypertrophy (70%), focal fibrosis on LGE (70%) and maximal hypertrophy at the basal anteroseptum (50%). With TTE, measured wall thickness was significantly lower (mean difference 2.3 ± 2.2 mm, *p* < 0.05), compared to CMR. TTE criteria for diagnosis of HCM were reported in only 50% of cases of new CMR diagnosis of HCM. The pattern of LVH on TTE was primarily concentric.

**Table 2:** Cox regression analysis for death and/or hospitalization with heart failure at minimum 6 month follow-up

	Univariate model, HR (95% CI)	*p*	Multivariate model, HR (95% CI)	*p*
*Demographics*				
Age	1.01 (0.99–1.05)	0.34		
Gender	1.48 (0.84–2.60)	0.17		
*Clinical Findings*				
Heart rate (b.p.m)	1.00 (0.98–1.02)	0.64		
Systolic blood pressure (mmHg)	1.00 (0.98–1.01)	0.38		
Diastolic blood pressure (mmHg)	0.97 (0.95–1.00)	0.03	0.99 (0.97–1.02)	0.48
NYHA III/IV	1.80 (1.02–3.17)	0.04	1.55 (0.83–2.89)	0.17
*Medical history*				
Hypertension	2.40 (0.58–9.87)	0.23		
Diabetes	1.03 (0.59–1.79)	0.91		
*Biochemistry*				
Sodium (mmol/L)	0.97 (0.90–1.05)	0.45		
Urea (mmol/L)	1.09 (1.02–1.15)	0.01	1.10 (1.01–1.21)	0.04
eGFR (ml/min per 1.73m^2^)	0.99 (0.97–1.00)	0.07	1.01 (0.99–1.03)	0.37
ZLog BNP (ng/L)	1.47 (1.08–2.01)	0.02	1.44 (1.03–2.02)	0.03
*CMR findings*				
New diagnoses group	1.75 (1.00–3.07)	0.05	1.92 (1.06–3.45)	0.03

Abbreviations are as for Table 1 and *HR* hazard ratio, *CI* confidence interval

### Constrictive pericarditis

Constrictive pericarditis was identified in 5 patients, with at least 3 out of the 4 main diagnostic parameters for CMR present in all cases (see Table 3). Whilst pericardial thickening on CMR was universally reported in patients with constrictive pericarditis, this finding was not identified in any of the TTE reports. Furthermore, in 3 out of 4 patients, TTE failed to identify septal bounce that was observed with CMR.

**Table 3:** Characteristics of newly diagnosed hypertrophic cardiomyopathy patients

Patient	Age	HTN	Image modality	Image grade	Maximal wall thickness		Hypertrophy pattern		SAM	LVOTO	LGE hyperenhancement		Likelihood of HCM
					mm	region	ASH	Concentric			Mid-wall	Insertion point	
A	71	+	TTE	2	15	Basal inferoseptum	–	+	–	–	n/a		Definite
			CMR	2	19	Basal anteroseptum	+	–	–	–	+	+	
^a^B	85	–	TTE	3	12	Apical septum	–	+	–	–	n/a		Definite
			CMR	3	10	Apical septum	–	–	–	–	–	–	
C	79	+	TTE	1	15	Basal inferoseptum	–	+	+	–	n/a		Probable
			CMR	2	15	Basal anteroseptum	+	–	–	–	–	+	
D	37	–	TTE	1	17	Basal inferoseptum	u/a	u/a	u/a	u/a	n/a		Definite
			CMR	3	22	Basal inferoseptum	–	+	–	–	+	+	
E	68	+	TTE	2	16	Basal inferoseptum	–	+	–	–	n/a		Definite
			CMR	2	21	Basal anteroseptum	+	–	–	–	+	–	
F	87	+	TTE	2	12	Basal inferoseptum	–	+	+	+	n/a		Probable
			CMR	3	15	Basal anteroseptum	+	–	+	+	+	+	
G	62	+	TTE	2	13	Basal inferoseptum	–	+	+	–	n/a		Probable
			CMR	2	15	Basal inferoseptum	+	–	+	–	–	–	
H	70	+	TTE	1	14	Basal anteroseptum	–	+	–	–	n/a		Probable
			CMR	2	15	Mid inferoseptum	+	–	–	–	+	–	
I	74	–	TTE	1	14	Basal anteroseptum	–	+	–	–	n/a		Probable
			CMR	3	17	Basal inferoseptum	–	+	–	–	–	–	
J	72	+	TTE	1	16	Basal anteroseptum	–	+	–	–	n/a		Probable
			CMR	3	18	Basal anteroseptum	+	–	–	–	+	–	

Abbreviations: TTE = transthoracic echocardiography; CMR = cardiac magnetic resonance; HTN = hypertension, LGE = late gadolinium enhancement imaging; u/a = unable to assess; n/a = not applicable; − = absent; + = present

Image grade: 1 = poor; 2 = fair; 3 = good

Diagnostic considerations for hypertrophic cardiomyopathy (HCM): LV wall thickness ≥ 15 mm, asymmetrical septal hypertrophy (ASH – septal: free wall thickness ratio > 1.3), apical HCM if apical wall thickness > 15 mm or apical:basal wall thickness ratio ≥ 1.3, left ventricular outflow tract obstruction (LVOTO) and systolic anterior motion of the mitral valve (SAM)

^a^Note: Patient B was diagnosed with apical HCM (spade-like configuration of the LV cavity and apical: basal wall thickness ratio ≥ 1.3)

### Clinical outcome

**Fig. 4:**
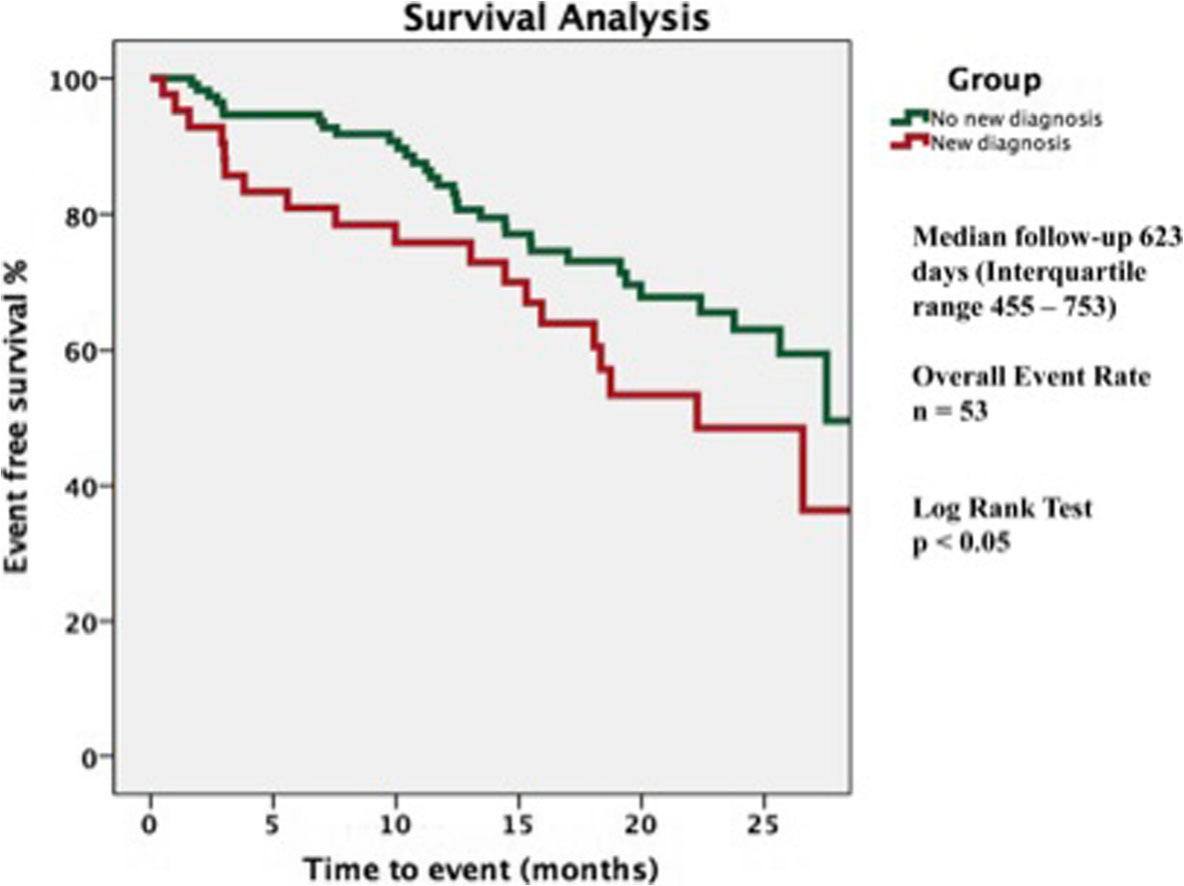
Kaplan Meier analysis for the composite endpoint of death and/or hospitalization with heart failure. Nil

During a median follow-up of 623 days (IQR 455–753), there were a total of 53 events (19 deaths, 34 hospitalizations with HF). Of these, ‘the new CMR diagnoses group’ accounted for 20 events (8 deaths, 12 hospitalizations with HF). Event-free rates (Fig. 4) were significantly lower in the ‘new CMR diagnoses’ group (52.4% vs 70.5%, log rank test: *p* < 0.05). The results of univariate and multivariate Cox proportional hazards analysis to predict events are shown in Table 4. On multivariate analysis, a new CMR diagnosis (hazard ratio [HR]: 1.92; 95% confidence interval [CI]: 1.06 to 3.45; *p* < 0.05), log BNP (HR: 1.44; CI: 1.03 to 2.02; *p* < 0.05, and urea (HR: 1.10; CI: 1.01 to 1.21; *p* < 0.05) were predictors of the primary endpoint.

**Table 4:** Imaging characteristics of newly diagnosed constrictive pericarditis patients

Patient	Image grade		Pericardial thickening		Pericardial effusion		Septal bounce		Septal E’ ≥ 9 cm/s	Pericardial enhancement
	TTE	CMR	TTE	CMR	TTE	CMR	TTE	CMR	TTE	CMR
A	2	2	–	+	+	+	–	–	–	+
B	2	3	–	+	–	+	–	+	+	+
C	1	3	–	+	–	–	–	+	+	+
D	1	2	–	+	–	+	–	+	+	–
E	1	3	–	+	+	+	+	+	–	+

Abbreviations: *TTE* transthoracic echocardiography, *CMR* cardiac magnetic resonance; − = absent; + = present

Image grade: 1 = poor; 2 = fair; 3 = good

## Discussion

The principal finding in our study is that stress CMR unmasks potentially clinically relevant undiagnosed cardiac pathology in a significant proportion of patients (27%) labelled as HFpEF after echocardiography. A clinically relevant proportion of our patients was identified as having hitherto unknown coronary artery disease or microvascular dysfunction. Moreover, despite being part of the TTE-based exclusion criteria at study entry, new cases of HCM and constrictive pericarditis were identified during subsequent CMR evaluation. Our observations suggest that previous intervention trials in HFpEF are likely to have included patients meeting one or more exclusion criteria, thereby possibly influencing treatment response. These additional pathologies, when grouped together in our cohort, were associated with adverse outcomes.

### ‘New CMR diagnoses’

The reasons for the higher pick-up rate of new clinical diagnoses with CMR are multiple. Firstly, the overall image quality for TTE in our study was poor, reflecting the clinical profile of our challenging population, with a high prevalence of obesity, lung disease and atrial fibrillation [21]. These comorbidities are typical of HFpEF as reported in the literature [1]. Furthermore, the low feasibility (inadequate endocardial border definition in nearly one-third) and diagnostic utility of TTE in HF has previously been reported and is subject to wider limits of agreement compared with CMR [9, 22]. The ability of CMR to interrogate any imaging plane and perform in vivo tissue characterization (e.g. by LGE) makes this the reference standard for detection of new diagnoses in our cohort [9–11].

Previous reports quote a wide range for the prevalence of CAD in HFpEF, comprising primarily data from epidemiological studies and registries. Furthermore, the presence of CAD was variably based on patient reporting, use of insensitive and non-specific investigations (e.g. ECG, exercise treadmill tests), inconsistent diagnostic cut-offs for angiographic disease severity, and did not incorporate CMR [23]. In this study, CMR increased the overall proportion of significant CAD (silent MI and/or ischaemia) from 21% to 34%, equivalent to a relative increase of 63%. These findings (and microvascular dysfunction) might be expected, given the proportion of elderly, hypertensive and diabetic patients in our cohort [24]. Furthermore, these greater number of ‘new’ CAD diagnoses is perhaps unsurprising given that CAD was not part of our exclusion criteria. We used a practical definition of HFpEF and current clinical guidelines [25] for HF do not mandate routine investigation for CAD unless accompanied by anginal symptoms recalcitrant to medical therapy. Additionally, the higher numbers of ‘silent’ CAD could also be explained by the inability of some patients to provoke clinical symptoms due to limited exercise capacity owing to co-morbidities. Conversely, exertional breathlessness may represent angina equivalent. The typical patterns of infarction (small number of segments and ≤50% transmurality) in our study are in keeping with overall preservation of LVEF. In such cases, the diagnostic accuracies of both ECG (Q wave) and TTE (RWMAs) are low in concordance with published literature [26].

Diagnosing HCM represents an imaging challenge in this cohort of patients. The latest HCM diagnostic guidelines [12] advocate a morphological description of imaging in suspected subjects. These guidelines are also more inclusive of considering HCM as a diagnosis in any patients whereby increased LV wall thickness cannot solely be explained by abnormal loading conditions. CMR features supportive of HCM in hypertensive patients include a more asymmetric pattern of LVH and LGE at the insertion points and in segments of maximal LV wall thickening [27, 28]. Furthermore, LGE is reportedly present in 65% with HCM, similar to our cohort [12].

HCM is characterized by non-specific diverse patterns of hypertrophy with or without left ventricular outflow tract obstruction or systolic anterior motion of the mitral valve [12, 14, 29]. In HFpEF, LVH is a common finding [1] and co-existing conditions such as ageing, obesity and hypertension are additional confounders [30]. Furthermore, hypertensive heart disease classically presents with concentric hypertrophy and wall thickness rarely exceeds 15–16 mm [28]. Deciphering the pattern of LVH according to mass and relative wall thickness calculations traditionally used in TTE is fraught with intrinsic methodological limitations [31]. These factors along with sub-optimal image quality [29] and the very high prevalence of hypertension (90%) may explain the underreporting of HCM by TTE in our cohort. In our study, patients who met wall thickness criteria for HCM on TTE were not reported as likely HCM most probably due to a predominant concentric pattern of LVH. Whilst TTE traditionally risks overestimating wall thickness (e.g. oblique cuts) [12], underestimation has been noted in a small (12%) proportion, especially if confined to the inferolateral, anterolateral or apical segments. In contrast, the superior endocardial definition afforded by CMR allows a more precise measurement of LV wall thickness and hypertrophy [29].

Current TTE diagnostic criteria for constrictive pericarditis have lower sensitivities compared to CMR (pericardial thickening: 36% vs 88%, septal bounce: 62% vs 81%) [5, 20]. In our cohort, the majority of these TTE parameters were not detected, which again is a likely reflection of poor image quality.

### Implications

Our CMR findings reinforce the marked clinical heterogeneity in HFpEF [1] and provide alternative explanations for symptoms in a significant minority of patients. Survival following silent MI is comparable to known MI [32]. Importantly, diagnosis by CMR enables initiation of effective secondary prevention treatment and guides revascularization, given that most affected myocardial segments identified in our cohort were viable [13]. Our data suggest that screening for significant CAD should be undertaken in patients with suspected HFpEF. A diagnosis of HCM has implications for both patients and relatives. CMR improves risk stratification and may enable earlier initiation of therapies such as implantable defibrillator devices [12]. Constrictive pericarditis is potentially curable and pericardial enhancement on LGE may predict treatment response [5].

### Implications for current HFpEF clinical trials

Our study has important implications and ramifications for HFpEF clinical trials and current treatment strategies. Variable definitions of HFpEF and phenotypic heterogeneity displayed in prior studies have previously been proposed as a key reasons for treatment failure [1, 3]. This has led to a paradigm shift in focus to study ‘purer’ subsets of HFpEF resulting in more detailed mechanistic studies. Our CMR study findings provide additional explanations for such poor outcomes whereby TTE remains the primary entry tool for trial enrolment. Our data suggests that TTE alone is incapable of rigorously excluding imaging phenocopies of HFpEF prior to study entry. Such conditions have alternate pathophysiological mechanisms, respond differently to existing therapies and contribute to adverse outcomes. While TTE is comparatively more extensively available, and therefore attractive for clinical trial design, access to CMR is rapidly increasing. Furthermore, CMR refines the diagnosis and sub-categorises HFpEF into ‘purer forms’ and alternative pathologies, enabling disease-specific tailored therapies, and provides prognostic data.

The routine use of stress CMR in HFpEF patients should refine diagnosis and treatment strategies as we move towards an era of precision medicine. However, further randomised trials are needed to assess the wider impact of CMR in terms of clinical outcome, resource utilization and cost-effectiveness.

### Limitations

The definition of HFpEF used in our study was not in accordance with current European Society of Cardiology (ESC) guidelines [8]. However, we took a pragmatic approach to reflect a real world setting. In particular, the presence of diastolic dysfunction was not a pre-requisite for study entry since recent contemporary clinical trials have highlighted normal diastolic function at rest in approximately a third of such patients [7]. Although all patients meeting inclusion criteria were invited, 26 out of 180 (14%) did not undergo CMR, which might raise concerns about its applicability to the wider HFpEF population. Whilst chronic obstructive pulmonary disease is quite prevalent in the clinical scenario of HFpEF, we only excluded patients with severe disease (and likewise severe valvular disease) to minimise the contribution from alternate causes of HF symptoms. Besides our cohort still comprised chronic obstructive pulmonary disease subjects in nearly one-fifth who underwent CMR. Six patients with pacemakers did not undergo CMR: at the time the study was conducted, our centre was not implanting CMR conditional devices. Although all CMR scans were performed solely at 3 T, we expect the study findings to be similar with a 1.5 T system.

Discriminating microvascular dysfunction from global coronary ischaemia can be challenging with CMR and raises the possibility of under-reporting of CAD. Furthermore, patients did not have stress echocardiography which may have identified more patients with ischaemia. In this cohort of patients with multiple risk factors for LVH, ultimately the imaging diagnosis of HCM is one of exclusion. However, the most recent ESC guidelines recommend defining HCM in patients with LVH ≥ 15 mm not solely explained by loading conditions [12]. Our CMR reports were generated using a clinical protocol exclusive of T1 and T2 mapping which were not routinely used at the time of study conduct. T1 mapping may have unmasked further hypertrophic phenotypes [12] such as cardiac amyloid and Anderson-Fabry’s disease, and T2 mapping may have been helpful in cases of constrictive pericarditis [5].

While the CMR reports were generated by GPM and ASHC, clinical endpoints were collated by PK who was not blind to CMR results. However, the HF hospitalization events were clearly objectively defined (see methods section) and assessment of vital status is robust. Some patients may have had hospitalizations exclusive of our hospital. However, there should be no systematic bias for those with or without ‘new’ diagnoses.

## Conclusions

In HFpEF, CMR identifies previously undetected pathology in a significant proportion of patients. This group of additional diagnoses is associated with worse outcomes and is an independent predictor of death and hospitalization due to HF.

## Abbreviations

ACEi: Angiotensin converting enzyme inhibitor
ARB: Angiotensin II receptor blocker
BNP: B-type natriuretic peptide
CAD: Coronary artery disease
CI: Confidence interval
CMR: Cardiovascular magnetic resonance imaging
ECG: Electrocardiogram
EF: Ejection fraction
eGFR: Estimated glomerular filtration rate
FEV_1_: Forced expiratory volume
FVC: Forced vital capacity
HCM: Hypertrophic cardiomyopathy
HF: Heart failure
HFpEF: Heart failure with preserved ejection fraction
HFrEF: Heart failure with reduced ejection fraction
HR: Hazard ratio
LGE: Late gadolinium enhancement imaging
LV: Left ventricle/left ventricular
LVEDVI: Left ventricular end-diastolic volume indexed to body surface area
LVEF: Left ventricular ejection fraction
LVESVI: Left ventricular end-systolic volume indexed to body surface area
LVH: Left ventricular hypertrophy
MI: Myocardial infarction
NYHA: New York Heart Association classification
RWMA: Regional wall motion abnormalities
TTE: Transthoracic echocardiography
